# Association of medical male circumcision and sexually transmitted infections in a population-based study using targeted maximum likelihood estimation

**DOI:** 10.1186/s12889-021-11705-9

**Published:** 2021-09-08

**Authors:** Lateef Amusa, Temesgen Zewotir, Delia North, Ayesha B. M. Kharsany, Lara Lewis

**Affiliations:** 1 Department of Statistics, School of Mathematics, Statistics and Computer Science, University of Kwazulu-Natal, Durban, South Africa; 2 Department of Statistics, University of Ilorin, Ilorin, Nigeria; 3 Centre for the AIDS Programme of Research in South Africa (CAPRISA), University of KwaZulu-Natal, Durban, South Africa; 4 School of Laboratory Medicine & Medical Sciences, Nelson R Mandela School of Medicine, University of KwaZulu-Natal, Durban, South Africa

**Keywords:** STI, Male circumcision, TMLE, IPTW, Full matching, South Africa

## Abstract

**Background:**

Epidemiological theory and many empirical studies support the hypothesis that there is a protective effect of male circumcision against some sexually transmitted infections (STIs). However, there is a paucity of randomized control trials (RCTs) to test this hypothesis in the South African population. Due to the infeasibility of conducting RCTs, estimating marginal or average treatment effects with observational data increases interest. Using targeted maximum likelihood estimation (TMLE), a doubly robust estimation technique, we aim to provide evidence of an association between medical male circumcision (MMC) and two STI outcomes.

**Methods:**

HIV and HSV-2 status were the two primary outcomes for this study. We investigated the associations between MMC and these STI outcomes, using cross-sectional data from the HIV Incidence Provincial Surveillance System (HIPSS) study in KwaZulu-Natal, South Africa. HIV antibodies were tested from the blood samples collected in the study. For HSV-2, serum samples were tested for HSV-2 antibodies via an ELISA-based anti-HSV-2 IgG. We estimated marginal prevalence ratios (PR) using TMLE and compared estimates with those from propensity score full matching (PSFM) and inverse probability of treatment weighting (IPTW).

**Results:**

From a total 2850 male participants included in the analytic sample, the overall weighted prevalence of HIV was 32.4% (*n* = 941) and HSV-2 was 53.2% (*n* = 1529). TMLE estimates suggest that MMC was associated with 31% lower HIV prevalence (PR: 0.690; 95% CI: 0.614, 0.777) and 21.1% lower HSV-2 prevalence (PR: 0.789; 95% CI: 0.734, 0.848). The propensity score analyses also provided evidence of association of MMC with lower prevalence of HIV and HSV-2. For PSFM: HIV (PR: 0.689; 95% CI: 0.537, 0.885), and HSV-2 (PR: 0.832; 95% CI: 0.709, 0.975). For IPTW: HIV (PR: 0.708; 95% CI: 0.572, 0.875), and HSV-2 (PR: 0.837; 95% CI: 0.738, 0.949).

**Conclusion:**

Using a TMLE approach, we present further evidence of a protective association of MMC against HIV and HSV-2 in this hyper-endemic South African setting. TMLE has the potential to enhance the evidence base for recommendations that embrace the effect of public health interventions on health or disease outcomes.

**Supplementary Information:**

The online version contains supplementary material available at 10.1186/s12889-021-11705-9.

## Background

Numerous public health initiatives to better control the incidence of HIV/AIDS and other sexually transmitted infections (STIs) have been implemented. One such public health intervention has been medical male circumcision (MMC), which focuses on the anatomical structure of the penis. It is well established that the inner foreskin of the penis is highly susceptible to infection and that the surgical removal of the foreskin, which is the retractable fold of tissue covering the head of the penis, reduces susceptibility to infections. Therefore, MMC is recognized as being one modifiable risk factor for STIs, including HIV in men. Evidence from three randomized controlled trials (RCTs) showed that MMC decreased heterosexual acquisition of HIV by 53 to 60%, herpes simplex virus type-2 (HSV-2) by 28 to 34% and genital ulcer disease among men [1–4]. Other studies [5–8], also found a protective effect of MMC against HIV infection and some sexually STIs acquired via heterosexual transmission.

The numerous studies highlighted above, amongst others, underline the importance of the relationship between MMC and the acquisition of STIs. However, these studies investigating the associations between MMC and STIs have estimated conditional effects, usually using traditional regression models. None of these studies has provided average or marginal treatment effects, usually estimated by RCTs and propensity score analyses. By mimicking an RCT, where a marginal treatment effect is obtained by contrasting the outcomes between the exposed and non-exposed groups, there is increasing interest in estimating marginal or average treatment effects using observational data, not conditional or adjusted treatment effects [9].

Besides estimating marginal treatment effects, model misspecification is another problem for assessing the association between the exposure (or treatment) and outcome. Misspecification of the model terms could substantially bias the estimated effects and the statistical inference [10]. Machine learning methods, using automated data-adaptive strategies that capture important patterns and interactions among variables, can typically overcome these limitations [11, 12]. Though machine learning has traditionally focused on risk prediction or classification, its utility has been extended to effect estimation and inference [13, 14]. Targeted maximum likelihood estimation (TMLE) is a doubly-robust semiparametric method that estimates exposure effects or associations without relying on model specifications [15]. It combines semiparametric estimation, using machine learning algorithms, with an additional estimation process to optimize a parameter of interest (e.g. risk difference, risk ratio, and odds ratio) [14].

This analysis investigates the associations between MMC and two STI outcomes with relatively high prevalence in the South African population. Specifically, we used a population-based study to estimate the association between MMC and HIV and HSV-2 among males in the KwaZulu-Natal province of South Africa. We obtained marginal prevalence ratios using TMLE and further compared our results with estimates from propensity score analyses, including propensity score full matching (PSFM) and inverse probability of treatment weighting (IPTW) methods.

## Methods

### Study design and participants

We used data from the HIV Incidence Provincial Surveillance System (HIPSS), a detailed and robust surveillance project that monitored HIV prevalence and incidence trends in a high HIV burden District in KwaZulu-Natal, South Africa. The HIPSS study aimed to assess the impact of programmatic intervention efforts, including HIV-related prevention and treatment programmes on HIV prevalence, uptake of antiretroviral therapy (ART), CD4 cell counts and viral suppression, in a real-world non-experimental setting. Survey weights that adjust for varying selection probabilities and differential non-response rates were included in the study design. The HIPSS study design, source population and recruitment procedures have been described previously [16, 17].

Briefly, HIPSS was a household population-based study conducted in the Vulindlela and the Greater Edendale areas in the uMgungundlovu district of KwaZulu-Natal, South Africa. The study had two cross-sectional surveys of randomly selected individuals aged 15–49 years, conducted 1 year apart. For each survey, a multi-stage cluster sampling method was used to choose enumeration areas, households and individuals. All participants who completed questionnaires had peripheral blood samples collected and were allocated a unique identification number, with a unique number allocated to link the household, respondents’ questionnaire and laboratory data [16, 17].

This study utilized the HIPSS household survey comprising 9812 men and women enrolled between June 2014 and June 2015. It had an overall individual participation rate of 69.1% among inhabitants of occupied households and 86.7% of enrolled households.

### Variables and inclusion criteria of participants

We included men who self-reported their MMC status and had reported being sexually experienced. The main exposure of interest was the MMC status, i.e. whether a participant had MMC or not. Those who reported being uncircumcised or traditionally circumcised (represents partial removal of the foreskin), or did not know their circumcision status, were classified as not having MMC. The two primary outcomes of interest in our analysis were the HIV test result (+ve = 1, −ve = 0) and HSV-2 test result (+ve = 1, −ve = 0). Venous blood samples were tested for HIV antibodies and antigens using the fourth-generation HIV enzyme immunoassays with the bioMérieux Vironostika Uniform II Antigen/Antibody Microelisa system (bioMérieux, Marcy-l’Étoile, France). The HIV 1/2 CombiRoche Elecsys (Germany) (Roche Diagnostics, Penzberg, Germany) and HIV-1 Western Blot Bio-Rad assay (Bio-Rad Laboratories, Redmond, WA, USA) were used to confirm positive samples. For measurement of HSV-2, serum was tested for HSV-2 antibodies via ELISA (HerpeSelect, Focus Diagnostics, Cypress, CA, USA). HIV and HSV-2 status were laboratory-derived; hence, they are not susceptible to self-report bias.

Covariates selected include age (in years), marital status (married, widowed/divorced/separated, single), education (no education, primary/ not completed high school, completed high school, degree/diploma), whether respondent drinks alcohol. Sexual behaviours include the number of lifetime sexual partners (1,2–5, 6+, refused to report), condom usage (always/sometimes, never), and whether the respondent had sex in the last 12 months. At the household level, we assessed total household monthly income (categorized) and whether the household receives social support grant. These variables are epidemiologically plausible or possible confounders for the relationship between MMC status and HIV/HSV-2 outcomes.

There is substantial evidence from the literature regarding the association amongst sexually transmitted infections, including HIV and HSV-2, with other STIs [18–21]. Thus, we further analyzed test results from the following STIs: *Chlamydia trachomatis, Trichomonas vaginalis*, syphilis, *Neisseria gonorrhoea, Mycoplasma genitalium* and hepatitis B.

From the original 3547 male participants, we removed 692 participants who reported never having had sex. We further excluded participants who had missing values for MMC status (*n* = 5). Our analytic sample consisted of 2850 male participants.

### Statistical analysis

We contrasted the marginally adjusted prevalence ratios of the HIV and HSV-2 outcomes that would be observed for the MMC exposure. In other words, we compared the prevalence for each of the two outcomes, when the men were medically circumcised with not being circumcised. Further, all contrasts were adjusted for important confounders including age, marital status, educational level, number of lifetime sexual partners, condom usage, sexual activity in the last 12 months, condom usage, alcohol use, coinfected with other STI, household monthly income, household receipt of social support grant. We estimated MMC associations with HIV and HSV-2 using TMLE, full matching on the propensity score (PSFM) and inverse probability of treatment weighting (IPTW).

**Fig. 1:**
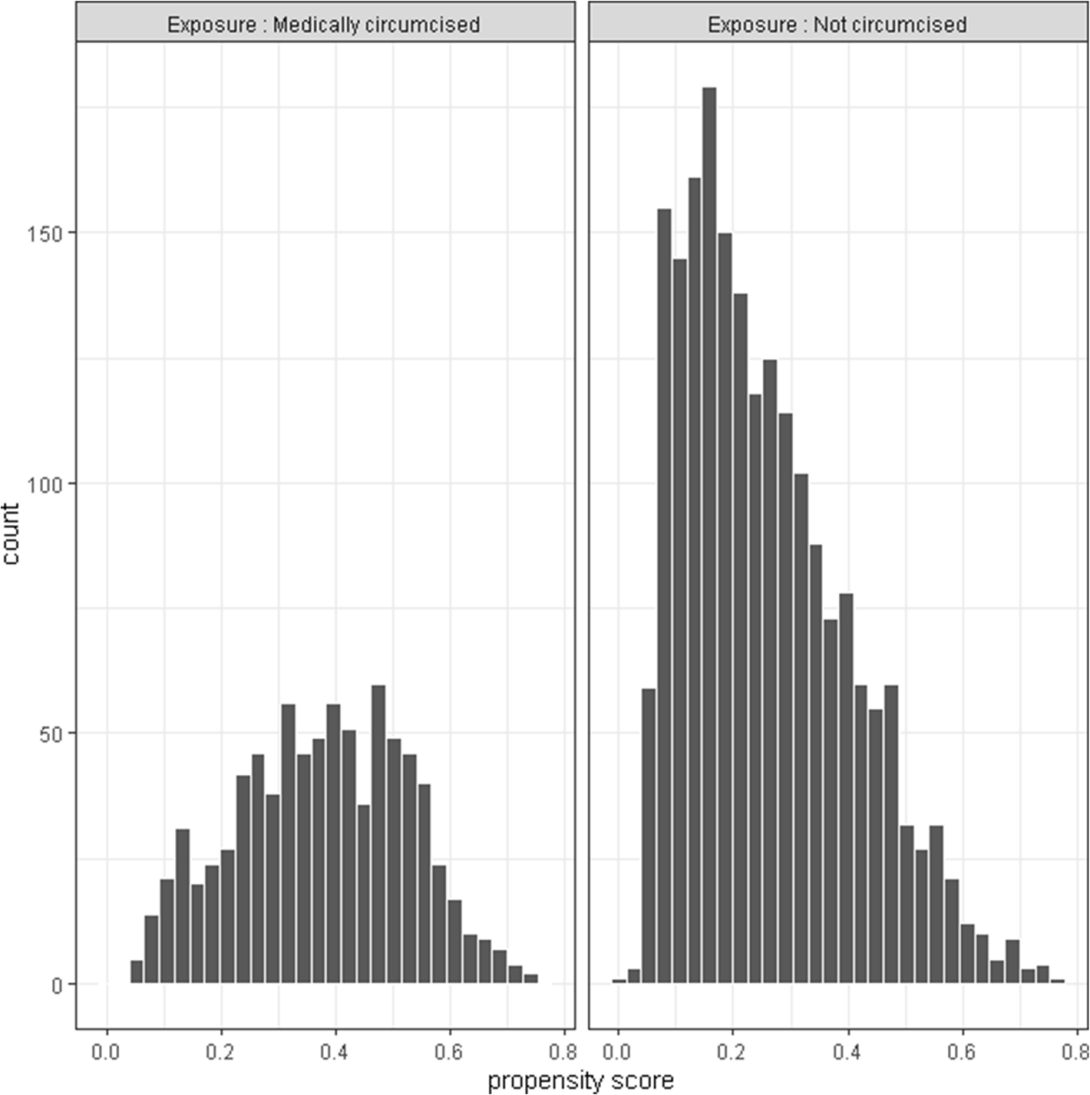
Histogram of estimated propensity score

Traditional circumcision is probably not equivalent from an STI risk perspective to being uncircumcised. However, in our analytic sample, the traditionally circumcised men were a small minority (4.5%) and had the same HIV and HSV-2 prevalence as the uncircumcised men. We conducted a sensitivity analysis to further understand the effect of including traditionally circumcised men in our uncircumcised group on our STI outcomes models (see Appendix Table 1 and Figure 1, Supplementary file 1). We dropped the traditionally circumcised men in our analytic sample and reran all our outcome analyses using the same methods described above for the main analyses.

We conducted a subgroup analysis to provide more novel information and establish heterogeneity in MMC association with HIV and HSV-2 (Table 3). The subgroup analysis was conducted on two subgroups of respondents: those who were coinfected with any other STI other than the primary STI outcome and those who were not coinfected with any other STI other than the primary STI outcome. This secondary analysis was necessitated because coinfections with other STIs might mediate associations observed for the primary outcomes. For the subgroup analysis, we used the same methods as described for the primary analyses.

### Targeted maximum likelihood estimation

Let *T*, *Y* denote the exposure (or treatment) indicator and observed outcome (MMC status and STI outcome, respectively, in this context), and let *W* be a vector including the identified confounders for the effect of *T* on *Y*.

The implementation of TMLE is straightforward. We first fitted an initial logistic regression of the STI outcome *Y*, given the MMC status and covariates, Q_0_ (*T, W) = E*_0_(*Y* | *T*, *W*). The estimate ${\mathrm{Q}}_n^0$ (*T*_*i*_, *W*_*i*_) and the predictions ${\mathrm{Q}}_n^0$ (1, *W*_*i*_) and ${\mathrm{Q}}_n^0$ (0, *W*_*i*_) were estimated with Super Learner. Super Learner is an ensemble learner of a pre-specified library of algorithms with parameters. It uses cross-validation to adaptively create an optimally weighted combination of estimates from candidate algorithms [22]. Optimality was defined based on each ensemble learner fit using 10-fold cross-validation, thereby reducing the chance of overfitting.

These estimates ${\mathrm{Q}}_n^0$ (*T*_*i*_, *W*_*i*_), ${\mathrm{Q}}_n^0$ (1, *W*_*i*_), and ${\mathrm{Q}}_n^0$ (0, *W*_*i*_) form additional columns in our data matrix. We then plugged-in our estimates ${\mathrm{Q}}_n^0$ (1, *W*_*i*_), and ${\mathrm{Q}}_n^0$ (0, *W*_*i*_) into our substitution estimator of the parameter of interest, prevalence (or risk) ratio, to obtain an untargeted estimate:

$$ {\psi}_{MLE,n}={Q}_n^0\left(1,{W}_i\right)/{Q}_n^0\left(0,{W}_i\right), $$

We next estimated the conditional distribution of MMC given covariates W, *g*_0 =_ *P* (*T | W*) with Super Learner, using the same set of algorithms. The predictions *g*_*n*_(1 |*W*_*i*_) and *g*_*n*_(0 |*W*_*i*_) were added to our data matrix. Initial estimates of Q_0_ (*T, W*) were then updated along a path of some fluctuation parameters, incorporating additional information from the propensity score function to reduce residual confounding in Q_0_ (*T, W*). This updating involves two steps: Firstly, *g*_*n*_, was used in a clever covariate ${H}_n^{\ast }$ (T, W) to define a parametric working model to fluctuate Q_0_ (*T, W*).

$$ {H}_n^{\ast}\left(\mathrm{T},\mathrm{W}\right)=\left(\frac{I\left(T=1\right)}{g_n\left(1|W\right)}-\frac{I\left(T=0\right)}{g_n\left(0|W\right)}\right) $$

For each individual with *T*_*i*_ =1 and *T*_*i*_ =0, the clever covariates are calculated as ${H}_n^{\ast }$ (1, *W*_*i*_) = $\frac{1}{g_n\left(1\ \right|{W}_i\Big)}$ and ${H}_n^{\ast }$ (0, *W*_*i*_) = $\frac{-1}{g_n\left(0\ \right|{W}_i\Big)}$, respectively. In addition to adding the columns ${H}_n^{\ast }$ (1, *W*_*i*_) and ${H}_n^{\ast }$ (0, *W*_*i*_), these values are then combined to form a column ${H}_n^{\ast }$ (*T*_*i*_, *W*_*i*_) in the data matrix.

In the second and final step, we estimated the fluctuation parameter ℇ_*n*_ by fitting an intercept-free logistic regression of Y on ${H}_n^{\ast }$ (T, W) with the logit of ${\mathrm{Q}}_n^0$ (T, W) being an offset (fixed quantity), where is the resulting coefficient of the clever covariate ${H}_n^{\ast }$ (T, W). We next updated the estimate ${\mathrm{Q}}_n^0$ into a new estimate ${\mathrm{Q}}_n^1$ of Q_1_ (*T, W*):

$$ \mathrm{Logit}\ {\mathrm{Q}}_n^1\left(\mathrm{T},\mathrm{W}\right)=\mathrm{Logit}\ {\mathrm{Q}}_n^0\left(\mathrm{T},\mathrm{W}\right)+{\varepsilon}_n\kern0.5em {\mathrm{H}}_n^{\ast}\left(\mathrm{T},\mathrm{W}\right) $$

We calculated

$$ \mathrm{Logit}\ {\mathrm{Q}}_n^1\left(\mathrm{T},\mathrm{W}\right)=\mathrm{Logit}\ {\mathrm{Q}}_n^0\left(1,\mathrm{W}\right)+{\varepsilon}_n\kern0.5em {\mathrm{H}}_n^{\ast}\left(1,\mathrm{W}\right) $$

for all individuals, and then

$$ \mathrm{Logit}\ {\mathrm{Q}}_n^1\left(0,\mathrm{W}\right)=\mathrm{Logit}\ {\mathrm{Q}}_n^0\left(\mathrm{T},\mathrm{W}\right)+{\varepsilon}_n\kern0.5em {\mathrm{H}}_n^{\ast}\left(0,\mathrm{W}\right) $$

for all individuals and included additional columns of ${\mathrm{Q}}_n^1$ (1, *W*_*i*_) and ${\mathrm{Q}}_n^1$ (0, *W*_*i*_) to our data matrix.

The updated estimates ${\mathrm{Q}}_n^1\left(1,W\right)$ and ${\mathrm{Q}}_n^1\left(0,W\right)$ were then used to compute the targeted estimator:

$$ {\psi}_{TMLE,n}={\mathrm{Q}}_n^1\left(1,{W}_i\right)\Big\}/{\mathrm{Q}}_n^1\left(0,{W}_i\right) $$

Our Super Learner library algorithms included generalized linear model (GLM), least absolute shrinkage and selection operator (LASSO) regularized GLM, generalized additive models, random forests, neural networks, k–nearest-neighbours, and the simple mean.

Full matching on the propensity score

Propensity score full matching (PSFM) is a synthesis of stratification on the propensity score (strata of the two exposure groups) and optimal pair-matching, which forms pairs of subjects from each of the two exposure groups such that the average within-stratum difference in the propensity score is minimized [23]. The stratification imposed by PSFM ensures that the following weighting system for estimating average treatment effects can be applied in each stratum for a subject *i*: ω_*i*_ = T_*i*_ P (T_*i*_ = 1) $\frac{\left(\mathrm{t}+\mathrm{u}\right)}{u}$ + (1 − T_*i*_) (1 – P (T_*i*_ = 1)) $\frac{\left(\mathrm{t}+\mathrm{u}\right)}{t}$ [24], where t and u denote the number of exposed and unexposed subjects in a given stratum, and P (T_*i*_ = 1) is the marginal probability of treatment in the overall sample.

Inverse probability of treatment weighting

We defined the propensity score as the conditional probability that a participant was exposed (or treated), given the covariates: e = P (*T* = 1 | *W*), estimated using the logistic regression. For IPTW, weights are computed to denote the inverse of the probability of receiving the treatment received by the subject. To obtain the IPT weights for estimating the average treatment effects, exposed (or treated) subjects are assigned a weight equal to the reciprocal of the propensity score, while the unexposed (or control) subjects are assigned a weight equal to the reciprocal of one minus the propensity score): ω_*i*_ = $\frac{{\mathrm{T}}_i\ \mathrm{P}\ \left({\mathrm{T}}_i=1\right)\ }{{\mathrm{e}}_i\ }$ + $\frac{\left(1-{\mathrm{T}}_i\right)\ \mathrm{P}\ \left({\mathrm{T}}_i=0\right)\ }{1-{\mathrm{e}}_i\ }$ [25].

For each of the two propensity score methods, these induced weights are then incorporated in a weighted univariate log-binomial regression model, which involves regressing the STI outcome on the MMC status to estimate the prevalence ratios. As suggested by Dugoff and colleagues [26], we also included the survey weight as an additional covariate in the propensity score model.

Analyses accounted for the survey design by incorporating the survey weights in their final estimation. Only a few variables had missing values and are shown in Table 1. In the multivariable analyses, ‘missing’ was made a separate category for the variable capturing the number of partners; for education level, missing values (*n* = 1) were excluded. TMLE was implemented using the *tmle* package [27] in R version 4.0.0. PSFM and IPTW were respectively implemented using the R packages *MatchIt* [28] and *WeightIt* [29].

**Table 1:** Descriptive characteristics of the male participants (*n* = 2850) in the HIPSS study according to their MMC status

Variable Description	Medically Circumcised	Not circumcised^a^
	(*N* = 830; 29.1%)	(*N* = 2020; 70.9%)
Age (years)	26.2 ± 7.5	31.8 ± 8.7
*Marital status*		
currently married	52 (6.3%)	187(11.1%)
widowed/divorced/separated	26 (3.2%)	108 (4.1%)
Single	752 (90.4%)	1725 (84.8%)
*Education (missing = 1)*		
primary school/incomplete high school	292 (37.3%)	1074 (56.2%)
completed high school	428 (50.9%)	783 (36.9%)
has a degree/diploma	91 (10.5%)	91 (4.1%)
No education	19 (1.3%)	71 (2.7%)
*Condom used with partner*		
always used condom	242 (31.2%)	402 (21.1%)
sometimes used condom	420 (47.4%)	1134 (52.4%)
never used condom	168 (21.5%)	484 (26.5%)
*Number of lifetime sex partners*		
1	141 (5.4%)	278 (9.9%)
2	126 (4.1%)	214 (8.1%)
3–5	263 (34.2%)	592 (30.4%)
6+	204 (25.3%)	552 (30.9%)
Refused to respond	96 (8.0%)	384 (13.2%)
*Total monthly household income*		
No income	105 (10.9%)	322 (12.9%)
≤ R2500	358 (40.8%)	1044 (48.2%)
R2500 - R6000	205 (27.6%)	373 (23.7%)
> R6000	93 (13.9%)	114 (7.9%)
No response	69 (6.7%)	167 (7.3%)
Household receives social support grant	389 (60.6%)	801 (55.7%)
Had sex in the last 12 months	703 (24.9%)	1650 (59.2%)
Drinks alcohol	378 (46.0%)	969 (48.0%)
*Neisseria gonorrhoea* (+ve)	13 (1.5%)	46 (2.0%)
*Chlamydia trachomatis* (+ve)	70 (8.8%)	99 (4.2%)
Syphilis (+ve)	17 (1.9%)	66 (3.2%)
*Mycoplasma genitalium* (+ve)	32 (4.0%)	154 (7.6%)
*Trichomonas vaginalis* (+ve)	24 (2.7%)	121 (5.4%)
Hepatitis B (+ve)	22 (2.5%)	127 (6.3%)
HSV-2 (+ve)	324 (36.4%)	1205 (59.8%)

Mean ± SD are reported for the continuous variable, age, while the other variables are represented as frequency (%). Reported statistics are population-weighted. ^a^ include those reporting not circumcised, do not know or traditionally circumcised

## Results

In the analytical sample of 2840 men, 29.1% reported receiving MMC. These medically circumcised men were younger and more likely to be unmarried than their uncircumcised counterparts. They also had a majority who had completed high school, used a condom with a recent sexual partner, not had sex in the last 12 months, had 3–5 sexual partners, their household earned less than 2500 Rands total income and received social support grant (Table 1).

To examine possible violations of the positivity assumption for estimators that rely on the propensity score, including the TMLE, we examined the estimated propensity score distribution. The histogram of the estimated propensity score by the exposure groups is shown in Fig. 1. As shown in Fig. 1, the lower tail of the distribution does not have tiny, close to zero, values in the exposed group (range: 0.056–0.751; median: 0.380). However, the minimal, near-zero values for the lower tail of the distribution of estimated propensity scores for the unexposed group (range: < 0.0001–0.765; median: 0.229) indicates near violation of the positivity assumption.

The prevalence of HIV and HSV-2 were lower among men who had received MMC than those who did not (Table 2). HSV-2 prevalence was higher (53.2%) than HIV (32.4%). Estimates of the unadjusted prevalence ratios showed a significant MMC association with each of the two STI outcomes.

**Table 2:** Prevalence and unadjusted prevalence ratios of the HIV and HSV-2 outcomes according to MMC status (*n* = 2850)

	HIV	HSV-2 ^a^
Medically circumcised prevalence; n (%)	139 (16.9%)	324 (36.5%)
Uncircumcised prevalence; n (%)	802 (38.8%)	1205 (60.1%)
Overall prevalence; n (%)	941 (32.4%)	1529 (53.2%)
PR (95% CI)	0.435 (0.350, 0.541)	0.609 (0.536, 0.692)

*PR* Prevalence ratio, *CI* Confidence interval, ^a^11 men were missing for HSV-2

**Fig. 2:**
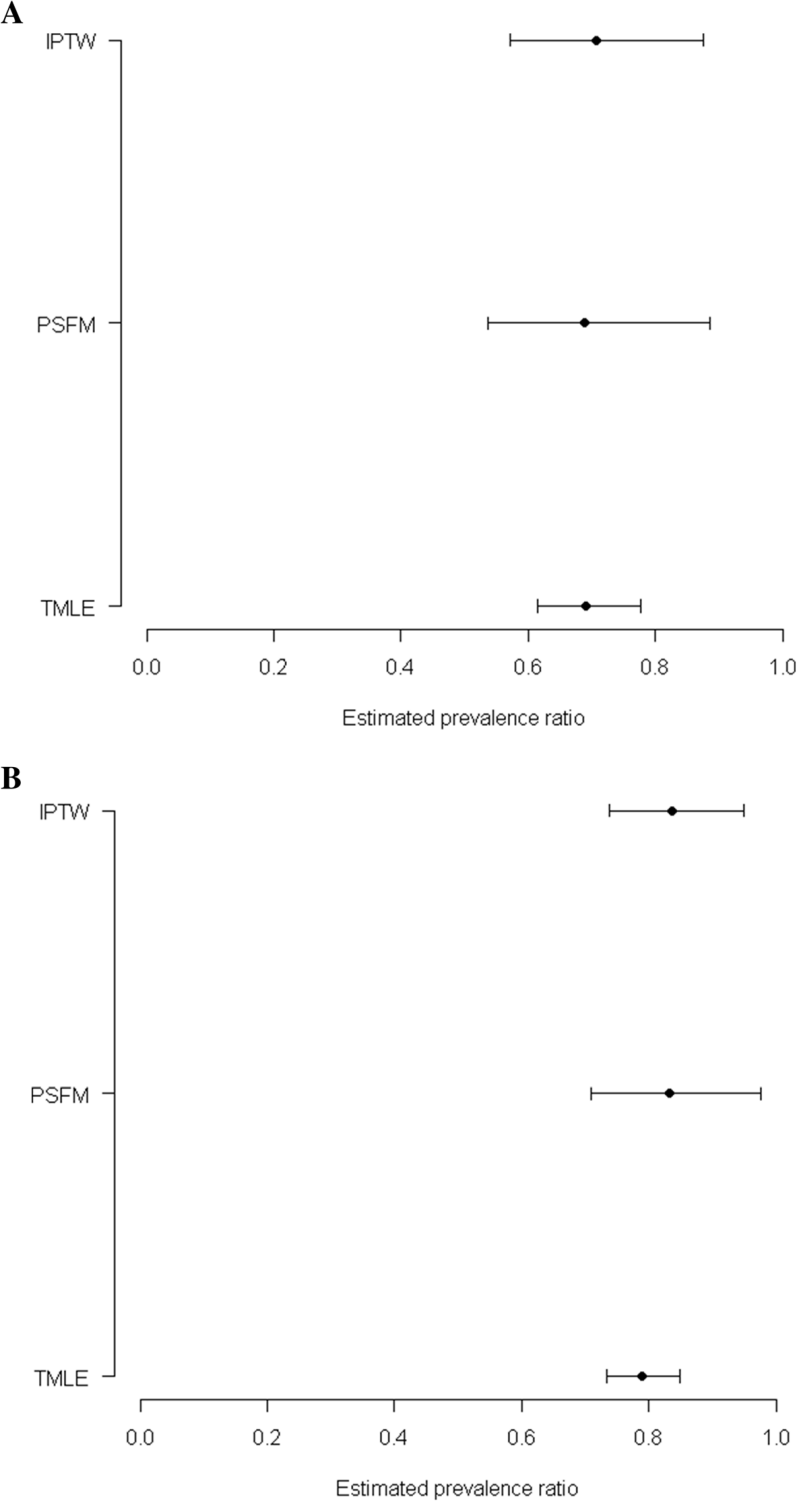
Associations between MMC and STI outcomes among men in the HIPSS study (*n* = 2850). **A** Adjusted prevalence ratios (95% CIs) of HIV among men who did MMC versus those who did not. **B** Adjusted prevalence ratios (95% CIs) of HSV-2 among men who did MMC versus those who did not. Note: 11 men were missing for HSV-2. Abbreviations: TMLE, Targeted maximum likelihood estimation; IPTW, Inverse probability of treatment weighting; PSFM, Propensity score full matching

After adjusting for the identified confounders, we found evidence of protective associations between MMC and HIV when the propensity score techniques were utilized (Fig. 2). For PSFM: HIV (PR: 0.689; 95% CI: 0.537, 0.885), and HSV-2 (PR: 0.832; 95% CI: 0.709, 0.975). For IPTW: HIV (PR: 0.708; 95% CI: 0.572, 0.875), and HSV-2 (PR: 0.837; 95% CI: 0.738, 0.949). Though the TMLE estimates were in the same direction as the estimates from the propensity score techniques, the TMLE estimates were more precise. For example, the TMLE estimates suggest that, for those who had MMC compared to the uncircumcised, the prevalence of HIV and HSV-2, was 46.9 and 20.5% lower, respectively (PR: 0.690; 95% CI: 0.614, 0.777) and HSV-2 (PR: 0.789; 95% CI: 0.734, 0.848).

For the Super Learner ensemble of algorithms, no single algorithm produced the best data fit, as measured by the lowest cross-validated mean squared error (results not shown). A further assessment of the weights (or coefficients) of each of the learning algorithm showed that only a subset of them contributed to the Super Learner predictions. For instance, in the Super Learner estimation of the relationship between HIV and MMC, generalized additive models contributed the most with a weight of 0.760, followed by random forest (0.223) and neural networks (0.013). The other algorithms had no contributions (weight = 0). A similar pattern was observed in the relationship between HSV-2 and MMC (results not shown).

Results from our sensitivity analyses did not meaningfully differ from our main analyses (See supplementary Appendix file). Our findings essentially remained the same in our subgroup analyses (Table 3) and did not suggest a non-protective association of MMC with HIV and HSV-2. Though for HSV-2, the multivariable analyses suggest a non-significant association, except for the TMLE estimate in the subgroup of those who were coinfected with any other STI. The protective association with HIV in the subgroup of those not coinfected with any other STI was relatively weakened, compared to those coinfected with any other STI.

**Table 3:** Sub-group analyses: Associations between MMC and STI outcomes among men in the HIPSS study

*HIV*	PR	95% CI		*HSV-2*	PR (95% CI)
Subgroup 1: Coinfected with at least one other STI					
unadjusted	0.624 (0.513, 0.760)	0.513	0.760	unadjusted	0.754 (0.657, 0.865)
TMLE	0.713 (0.630, 0.807)	0.630	0.807	TMLE	0.916 (0.849, 0.987)
PSFM	0.763 (0.609, 0.956)	0.609	0.956	PSFM	**0.896 (0.737, 1.090)**
IPTW	0.770 (0.629, 0.943)	0.629	0.943	IPTW	**0.902 (0.808, 1.008)**
Subgroup 2: Not Coinfected with any other STI					
unadjusted	0.256 (0.109, 0.599)	0.109	0.599	unadjusted	0.707 (0.574, 0.869)
TMLE	0.419 (0.248, 0.709)	0.248	0.709	TMLE	**1.042 (0.918, 1.183)**
PSFM	0.272 (0.097, 0.763)	0.097	0.763	PSFM	**0.879 (0.678, 1.141)**
IPTW	0.309 (0.143, 0.666)	0.143	0.666	IPTW	**0.887 (0.705, 1.115)**

*Abbreviations*: *PR* Prevalence ratio, *TMLE* Targeted maximum likelihood estimation, *IPTW* Inverse probability of treatment weighting, *PSFM* Propensity score full matching

11 men were missing for HSV-2. Bolded PRs were not significant at the 5% level

## Discussion and conclusion

We examined the utility of a relatively new methodology, targeted maximum likelihood estimation technique (TMLE), to estimate the association between MMC and sexually transmitted infections among males in a South African population-based study. This study adds to the body of growing knowledge providing evidence of the benefits of MMC in STI prevention. Specifically, we found that MMC has a protective association with HIV and HSV-2. Though the utilization of TMLE did not indicate a null effect nor alter the direction of the association, we found evidence of more precise effects.

The utilized HIPSS study also collected data on other STIs like *Chlamydia trachomatis, Neisseria gonorrhoea, Mycoplasma genitalium, Trichomonas vaginalis*, syphilis and hepatitis B infection. However, we selected HIV and HSV-2 because of their relatively high prevalence compared to other STIs, and the application of TMLE for rare outcomes is still in its infancy [30]. Moreover, previous reports [1] have shown that MMC associations with STIs other than HIV and HSV-2 were unreliable as the study was underpowered to detect rare STI outcomes.

Public health interventions for HIV and HSV-2 are critically important to study, especially in African settings with high burden syndemics. With over 7 million HIV positive individuals, South Africa has the highest number of people living with HIV globally. The KwaZulu-Natal province is the worst hit, with a prevalence of 27% as recorded at the end of 2017 [31]. There was an estimated 417 million cases of HSV-2 globally in 2012 [32]. The currently reported prevalence of HSV-2 in sub-Saharan Africa is as high as 80% among men and women aged 35 and older [33]. Biological and epidemiological evidence further suggests a cofactor effect of HIV and HSV-2. In other words, HSV-2 infection increases the likelihood of HIV acquisition [34, 35].

Parametric models require the correct specification of the functional form of the relationship between the exposure and the confounders or the outcome-confounders relationship. This requirement is challenging and not usually satisfied in practice. The most attractive and unique property of TMLE is its double-robustness, which reduces bias due to model misspecification [14]. This doubly-robust property ensures that TMLE estimates are unbiased if either the exposure or outcome model is consistently estimated. TMLE, like other doubly-robust techniques, offers an opportunity to rely on nonparametric methods (like machine learning) in its estimation process, thereby increasing efficiency [15]. Previous theoretical and simulation studies have shown that TMLE has greater efficiency and less bias when compared with misspecified parametric and nonparametric singly robust methods [14, 36]. This was also evident from the result of our TMLE estimates and confidence intervals in this study.

The proportion of refusals or non-participation of the utilized HIPSS study at the household and individual levels was lower than most community-based surveys [37]. Although the utilized data source is robust, it is cross-sectional. Thus, we are limited by the ability to conclude the temporal relationship between the self-reported factors with the STI outcomes. In other words, it cannot be determined whether observed associations existed before the STI outcomes or vice-versa. Data other than from the STI outcomes came from self-reports; hence, our study is likely to suffer from self-recall bias due to differential recall or social desirability. Since we didn’t assess circumcision status by clinical examination, there is a risk of classification bias, which could bias the estimated effect towards the null. In further studies of HIV incidence, validating self-reported circumcision status by clinical examinations may clarify the impact of such bias.

Not controlling for important risk factors such as a history of narcotics usage and additional comorbidities, which were not in the HIPSS database, is another limitation of this study.

For HIV, we did not exclude key subpopulations whose likelihood of acquisition would not result from heterosexual transmission. Our inclusion of these subpopulations would most likely bias associations towards the null since there will be less impact of their circumcision status on their HIV acquisition risk. Most of these limitations will be partly addressed by a planned analysis of a longitudinal cohort study capturing STI incidence, thereby validating findings from this study and others that have utilized the HIPSS study.

Our results provide further evidence of the protective association of MMC against HIV and HSV-2 in men. This study has important practical implications for studies using nonparametric estimation techniques. Notably, TMLE estimates should be interpreted in light of a careful assessment of the propensity score distribution among the exposed and unexposed and results from alternative parametric and nonparametric techniques. Due to its double robustness, TMLE, compared to its competitors, often results in efficiency gains and bias reduction of estimated exposure effects. In general, the TMLE method can advance epidemiology and public health, enhancing the evidence base for recommendations that embrace the effect of public health interventions on health or disease outcomes.

## Supplementary Information

**Additional file 1: Appendix Table 1.** Sensitivity analyses: Prevalence and unadjusted prevalence ratios of the HIV and HSV-2 outcomes according to MMC status (*n* = 2719). **Appendix Figure 1.** Sensitivity analyses: Associations between MMC and STI outcomes among men in the HIPSS study (*n*=2719).

## Data Availability

The dataset used in this study is available from the corresponding author upon reasonable request.

## Abbreviations

HIV: Human immunodeficiency virus
STI: Sexually transmitted infection
HSV-2: Herpes simplex virus type-2
MMC: Medical male circumcision
TMLE: Targeted maximum likelihood estimation
IPTW: Inverse probability of treatment weighting
PSFM: Propensity score full matching
