# Supplementary material for: Association of medical male circumcision and sexually transmitted infections in a population-based study using targeted maximum likelihood estimation
Source: BMC Public Health. 2021 Sep 8;21:1642. doi: 10.1186/s12889-021-11705-9 (PMC8425067; doi:10.1186/s12889-021-11705-9)
Supplement: Supplementary file 1 — Additional file 1: Appendix Table 1. Sensitivity analyses: Prevalence and unadjusted prevalence ratios of the HIV and HSV-2 outcomes according to MMC status (n = 2719). Appendix Figure 1. Sensitivity analyses: Associations between MMC and STI outcomes among men in the HIPSS study (n=2719). [file 12889_2021_11705_MOESM1_ESM.docx]

**Appendix Table 1.** Sensitivity analyses: Prevalence and unadjusted prevalence ratios of the HIV and HSV-2 outcomes according to MMC status (n = 2719)

|  | HIV | HSV-2 ^a^ |
| --- | --- | --- |
| Medically circumcised prevalence; n (%) | 139 (16.9%) | 324 (36.5%) |
| Uncircumcised prevalence; n (%) | 754 (38.9%) | 1119 (59.2%) |
| Overall prevalence; n (%) | 941 (32.4%) | 1529 (53.2%) |
| PR (95% CI) | 0.434 (0.349, 0.540) | 0.615 (0.541, 0.699) |

Prevalence ratio (PR); CI: Confidence interval; ^a^11 men were missing for HSV-2

**A**


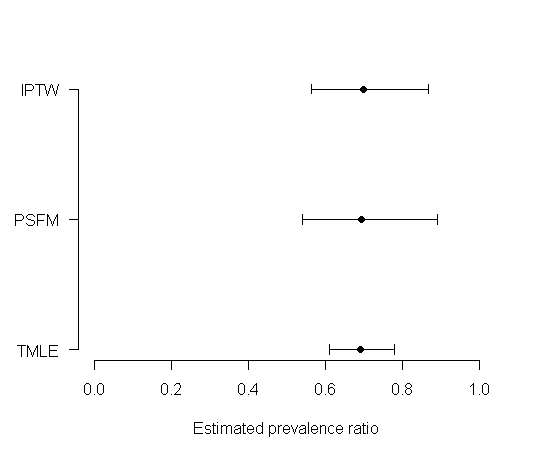


**B**


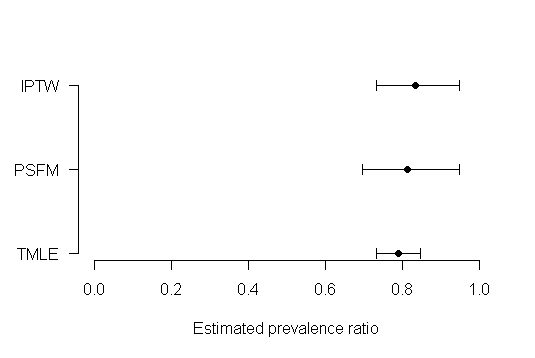


**Appendix Figure 1.** Sensitivity analyses: Associations between MMC and STI outcomes among men in the HIPSS study (n = 2719).

**Legend:** (A) Adjusted prevalence ratios (95% CIs) of HIV among men who did MMC versus those who did not. (B) Adjusted prevalence ratios (95% CIs) of HSV-2 among men who did MMC versus those who did not.

Note: 11 men were missing for HSV-2.

Abbreviations: TMLE, Targeted maximum likelihood estimation; IPTW, Inverse probability of treatment weighting; PSFM, Propensity score full matching.
